# In vivo estimation of motor unit intrinsic properties in individuals with spinal cord injury

**DOI:** 10.1186/s12984-025-01659-z

**Published:** 2025-06-05

**Authors:** Zhihao Duan, Asta Kizyte, Emelie Butler Forslund, Elena M. Gutierrez-Farewik, Pawel Herman, Ruoli Wang

**Affiliations:** 1 Department of Engineering Mechanics, KTH MoveAbility, KTH Royal Institute of Technology, Stockholm, 100 44 Sweden; 2 Department of Neurobiology, Care Science and Society, Karolinska Institutet, Stockholm, 141 83 Sweden; 3 Aleris Rehab Station R&D Unit, Solna, 169 89 Sweden; 4 Department of Women’s and Children’s Health, Karolinska Institutet, Stockholm, 171 77 Sweden; 5 Division of Computational Science and Technology, Electrical Engineering and Computer Science, KTH Royal Institute of Technology and Digital Future, Stockholm, 100 44 Sweden

**Keywords:** HD-EMG decomposition, Motor neuron Spike trains, Motor neuron modelling, Soma size, Discharge rate

## Abstract

**Background:**

Individuals who have experienced spinal cord injury (SCI) may exhibit various muscle-related neurophysiological adaptations, including alterations in motor unit (MU) size and firing behavior. However, due to the technical challenges of in vivo measurement, our understanding of the alterations in the electrophysiological parameters of these MUs remains limited. This study proposed an integrated approach using high-density electromyography (HD-EMG) decomposition and motor neuron (MN) modelling to estimate the intrinsic properties of MUs in vivo and investigated alterations of these properties in persons with SCI.

**Methods:**

HD-EMG signals were recorded during submaximal isometric dorsiflexion and plantar flexion tasks on tibialis anterior (TA), soleus, and gastrocnemius medialis muscles from twenty-six participants with SCI and eighteen non-disabled controls. The HD-EMG signals were subsequently decomposed into MN spike trains and the common synaptic input to the MN pool was estimated. A simplified leaky integrate-and-fire neuron model was then used to simulate MN spiking trains, with soma size and inert period as tunning parameters, which are crucial for MU recruitment and firing patterns, respectively. These parameters were estimated by fitting the instantaneous discharge frequencies of decomposed and simulated spike trains via a genetic algorithm.

**Results:**

The results showed a prolonged inert period in the TA of the persons with SCI. This finding suggested that the MUs in the TA have a slower recovery period before becoming excitable again, which may result in a lower firing rate of MUs in the TA muscle. No significant differences were observed in the soleus and gastrocnemius medialis muscles between the SCI and control groups for either the soma size or inert period parameters.

**Conclusions:**

The simplified leaky integrate-and-fire model exhibited robustness in estimating MN parameters in vivo, offering valuable insights into personalized MU behavior monitoring. To the best knowledge of authors, this is the first study to combine HD-EMG and MU modeling to investigate MU electrophysiological changes in persons with SCI in vivo. This novel approach offers a comprehensive understanding of MU properties adaptations following neurological disorders and informs the development of novel rehabilitation strategies.

**Supplementary Information:**

The online version contains supplementary material available at 10.1186/s12984-025-01659-z.

## Introduction

Motor units (MUs), consisting of a motor neuron (MN) and the muscle fibers it innervates are fundamental units for force generation and modulation of skeletal muscles. The central nervous system modulates muscle force production through the transformation of common synaptic inputs to the pool of MNs. MNs receive synaptic inputs from various sources, which are then integrated to determine the firing patterns and recruitment of different-sized MUs. After spinal cord injury (SCI), damage to the spinal cord leads to the disruption of neuronal axons, the death of neurons as well as dysfunction of peripheral MNs, often resulting in muscle weakness, spasticity, and poor motor control [1]. Studies have extensively explored the effects of SCI on muscle atrophy, muscle weakness, and disrupted muscle coordination [2, 3]. However, limited knowledge exists in MU adaptation after SCI due to the challenges of direct measurement in vivo. MU firing patterns are crucial for producing controlled and sustained muscle contractions. Wiegner et al. [4]. found that SCI can lead to a lower average firing rate of single MUs in the biceps brachii and tibialis anterior (TA) muscles, leading to muscle weakness by altering the frequency and coordination of action potentials. Similarly, MU size can be affected after SCI through the loss of MNs and subsequent reorganization within the motor pool. Li et al. reported that incomplete SCI can lead to a significantly reduced number of MUs across hand muscles, with an observable increase in the MU sizes in the first dorsal interosseous and hypothenar muscles compared to controls [5]. This study suggested that MU reorganization may contribute to weakened hand function in persons with SCI. Despite these findings, several critical gaps remain in quantifying intrinsic properties of MUs e.g., MU size and firing patterns in humans in vivo and understanding their adaptation following a neurological impairment. Accessing these fundamental electrophysiological parameters requires specialized neurophysiological techniques, such as isolating and recording the activity of individual MU using fine wire electrodes, which are not feasible to implement in clinical populations such as persons with SCI. Further research is therefore needed to develop non-invasive methods for assessing the intrinsic electrophysiological properties of MUs.

Surface electromyography is the most common method to study electrophysiological properties of muscle contraction, while high-density electromyography (HD-EMG) provides additional possibilities to quantify the firing behavior of MUs in vivo. Decomposition methods, based on convolutive blind source separation [6] or FastICA [7], can be used to separate individual MU activities and discharge timings from HD-EMG signals. HD-EMG, combined with decomposition methods, has been used to analyze MN discharge characteristics, i.e. MU firing rate and MU recruitment threshold [8, 9] and to track MU firing patterns such as temporal dynamics of MU activity and the evolutionary trajectory of motor control over the lifespan [10, 11]. Moreover, this advanced technique has also been applied to study MU behavior alteration following neurological impairment. It has been used to detect reduction in MU firing rates within the affected muscle groups in individuals with chronic stroke, providing MU-level insights into observed muscle weakness and coordinated movement difficulties [12–14]. Despite the extensive application of HD-EMG in studying MU behavior, knowledge remains limited on how the intrinsic electrophysiological properties of MUs change following neurological impairments, i.e., SCI and how these changes impact MU behavior.

Spike models that incorporate key electrophysiological properties such as membrane resistance, capacitance, and voltage threshold can be used to simulate the generation and propagation of MN spike trains and capture their firing patterns in response to synaptic inputs. Biophysically detailed compartmental spike models such as the Hodgkin-Huxley model [15] can well approximate the dynamics of single neurons by simulating the complex interplay of ionic currents and membrane dynamics. Rafael et al. have used a single-compartment conductance-based model to estimate intrinsic MN parameters and simulate their firing characteristics in able-bodied persons [16, 17]. This approach enabled the creation of subject-specific in silico MN pools that accurately predict in vivo MN firing patterns. However, the high dimensionality and intricate structure of the Hodgkin-Huxley model result in many tunning variables, thus increasing computational costs. Moreover, the Hodgkin-Huxley model, which incorporates various electrophysiological channel-related parameters to simulate the characteristics of MN pools, faces challenges in accurately estimating MN pool firing patterns due to a lack of sufficient empirical data in mammals [18]. In contrast, the leaky integrate-and-fire (LIF) model simplifies the process by focusing on key neuron membrane dynamics with fewer parameters, effectively capturing essential aspects such as firing patterns. Moreover, electrophysiological parameters in the LIF model have been quantitatively examined in mammalian experiments, revealing their interconnections, and established mathematical relationships [19]. This streamlined approach not only reduces computational demands but also enables more realistic and robust representations of MN pool characteristics, supporting more accurate modeling of overall MN behavior.

HD-EMG decomposition enables the identification of individual MU spike trains, offering valuable insights into MU firing patterns and coordination. While MU spike trains provide information on temporal activation and recruitment, they do not directly reveal the intrinsic electrophysiological properties of MNs, such as neuron size, excitability, and their recovery mechanisms. To bridge this gap, we propose an integrated pipeline that combines HD-EMG decomposition with MN modeling to estimate physiologically meaningful parameters reflecting the intrinsic properties of MUs in vivo. This approach extends the analysis beyond observed MU firings to infer underlying physiological mechanisms governing MU recruitment and firing behavior. Applying this method, we investigate potential alterations in these parameters in persons with SCI and compare them to a non-disabled cohort, providing a more comprehensive understanding of neuromuscular function and motor control adaptations after SCI.

## Methods

This is a sub-study of a larger collaborative project between Promobilia MoveAbility Lab at the KTH Royal Institute of Technology, Karolinska Institutet and Aleris Rehab Station, Stockholm, Sweden described in detail earlier [20]. Participants with SCI were a subset of those originally recruited for a study by Butler Forslund et al. [20]. Participants were selected based on the following inclusion criteria: (1) incomplete paraplegia for at least one year or considered neurologically stable; (2) aged 18 to 75 years; (3) ability to walk independently for at least 30 m with or without walking aids, but without help from another person, and habitually walking in daily life; (4) having less than full muscle strength but sufficient capacity to complete the test procedures. Exclusion criteria included: (1) no neurological sequelae following SCI; and (2) individuals who do not walk regularly in their daily life or only walk during training sessions. All participants with SCI included in this study had incomplete spinal cord injuries, and all references to SCI in the manuscript pertain to this subgroup. Participants with incomplete SCI who matched the inclusion criteria were recruited consecutively upon scheduled SCI follow-up appointments to the specialized SCI clinic Spinalis at Aleris Rehab Station in Stockholm, Sweden.

### Participants

Data from twenty-three participants with sub-acute and chronic SCI were included (thirteen males; age 55.2 ± 12.8 years; height 173.1 ± 9.1 cm; weight 80.30 ± 17.69 kg). The median time since injury was 65 months, with an interquartile range of 111 months. They were all classified as paraplegia with incomplete SCI AIS D according to the American Spinal Injury Association scale, indicating that all had normal function in upper extremities but varying extents of impaired motor and/or sensory function in lower extremities and trunk. The injury level was as follows: 11 participants with thoracic (T1-T6), 7 with thoracic (T7-T12), 3 with lumbar, and 2 with cervical SCI (only minor sensory deficits on cervical level). Eighteen non-disabled participants (seven males; age 53.4 ± 12.1 years; height 169.7 ± 8.6 cm; weight 69.0 ± 12.9 kg) with no known neurological disorders or recent lower limb injuries were recruited for the study. The study was approved by the Swedish Ethical Review Authority (2020–02311). All participants signed an informed consent after receiving oral and written information. The data collection was performed at the Promobilia MoveAbility Lab at KTH Royal Institute of Technology (Stockholm, Sweden).

### Experimental protocol and data collection

The participants were seated comfortably on a chair with one leg securely fastened to a stationary isometric dynamometer (OT Bioelettronica, Turin, Italy, sampling frequency 100 Hz) with the knee flexed at 90° and ankle joint at 0°. For participants with asymmetrical motor function, measurements were taken on the side with weaker muscles. For those without motor function asymmetry, measurements were conducted on the side with the most reduced sensory function. For control participants, measurement was performed on a randomly selected side. Participants were first instructed to perform maximal isometric voluntary contraction of ankle dorsiflexors and plantarflexors for 3 sec. This procedure was repeated twice with one minute rest between trials, and the maximal voluntary contraction (MVC) was defined as the maximum value obtained from the two repetitions. Subsequently, participants were asked to perform submaximal isometric contraction following a trapezoidal torque profile guided by a visual cue. Each torque profile included a 5-second ramp-up, a 4-second plateau at 20% or 50% MVC, and a 5-second ramp-down, with the order of MVC levels randomized. Each trial, either in dorsiflexion or plantar flexion comprised four repetitions of the trapezoidal profile with a 10-second rest in between each repetition.

The torque was recorded by the dynamometer with an S-beam bidirectional load cell and a single-channel general-purpose amplifier (Forza, OT Bioelettronica). HD-EMG signals were recorded using three electrode grids: a 32-channel grid (GR10MM0804, OT Bioelettronica) above the soleus (SOL) muscle, two 64-channel grids (GR08MM1305 and GR10MM0808, OT Bioelettronica) placed above the TA and gastrocnemius medialis (GM), respectively. The skin was first shaved and cleansed using an abrasive pad and alcohol. Electrode grids were then attached using adhesive foam filled with conductive paste following SENIAM guidelines for proper grid placement [21]. An elastic band was placed over the electrodes with slight tension to maintain consistent skin contact throughout the experiment. Additionally, strap electrodes dampened with water were placed around the wrist (serving as the ground electrode), knee (serving as the reference electrode for SOL), and ipsilateral ankle (serving as the reference electrode for GM and TA). The HD-EMG signals were recorded in monopolar mode and band-pass filtered between 10 Hz and 500 Hz. Both torque and HD-EMG signals were digitized at a sampling rate of 2048 Hz using a multichannel acquisition system (EMG-Quattrocento, OT Bioelettronica).

### HD-EMG decomposition

**Fig. 1:**
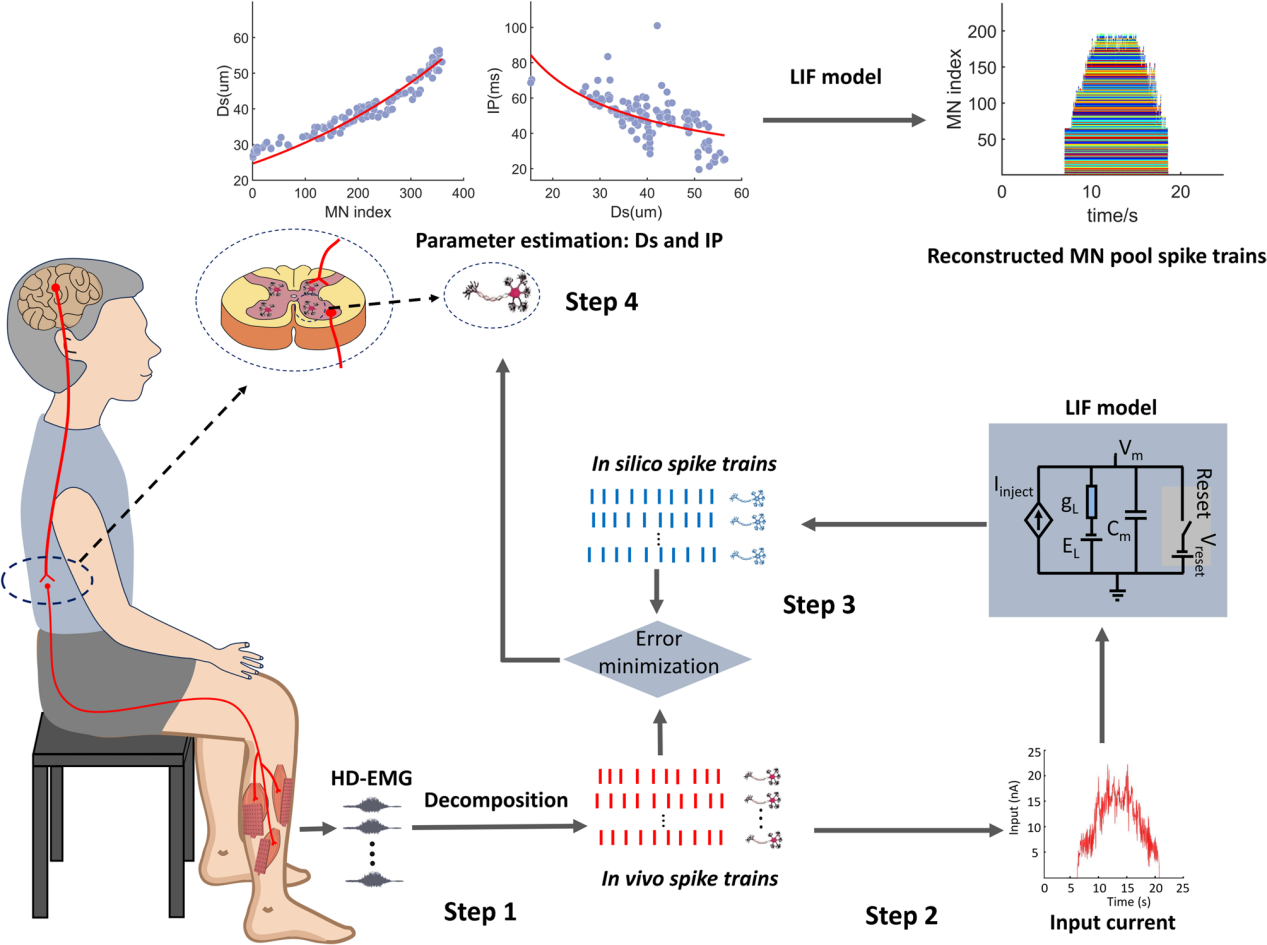
Overview of MN parameter estimation framework. The HD-EMG signals from the TA, SOL, and GM muscles were collected and decomposed into spike trains using the convolution kernel compensation blind source separation algorithm. The input current was estimated based on the decomposed spike trains. The LIF model was then implemented to generate in-silico spike trains. The MN parameters Ds and IP were estimated by matching the individual spike train decomposed from HD-EMG signals with the spike train generated by the LIF model. After obtaining these MN parameters, their distributions across the entire MN pool were modelled, and pool spike trains were generated to represent the spiking activity within the pool

The HD-EMG signals underwent band-pass filtering between 20 Hz and 500 Hz using a second-order Butterworth filter and subsequently were decomposed using the convolutive blind source separation method [6, 22] (DEMUSE software, version 6.0; University of Maribor, Slovenia) to extract individual MN spike trains. The decomposed spike trains were then visually inspected and manually edited in accordance with the guidelines outlined in the manual [23]. Only spike trains with a pulse-to-noise ratio > 30 dB were included for further analysis, ensuring a sensitivity exceeding 90% and a false-positive rate below 2% [24]. The schematic workflow was illustrated in Fig. 1, consisting of four key steps: [1] decomposing HD-EMG signals into individual MN spike trains [2]. Estimating the common synaptic input (CSI) driving the MN pool [3] Simulating in silico spike trains using a simplified LIF model to match the recruitment and discharge frequencies of the decomposed MN spike trains, thereby estimating MN model parameters [4] Using optimized parameters to infer their distribution within the MN pool.

### CSI estimation

The cumulative spike train was first computed by accumulating all individual MN spike trains obtained from the decomposition into single population spike train. The CSI, representing the net drive to the MN pool of one single muscle, was then estimated by applying a low-pass filter to the cumulative spike train within the bandwidth of 0–10 Hz. It has been shown that this frequency range is critical for force generation because it reflects the common input to the MN pool and serves as the primary determinant of force production [25–27]. The MN pool functions as a filter for independent synaptic noise received by individual MNs, linearly transmitting the CSI to modulate spike generation across the pool. Consequently, the final cumulative input current *I(t)*, responsible for spike generation of the MN pool, was determined as a linear function of CSI, with a constant gain *G* uniformly across the MN pool (Eq. 1) [28].

1 $$I(t)=\left\{ {\begin{array}{*{20}{c}} {0{\text{ if }}t<{T_1}} \\ {{I_1}+G \cdot \operatorname{CSI} (t)} \end{array}{\text{ with }}G=\frac{{{I_N} - {I_1}}}{{{T_N} - {T_1}}}} \right.$$

where *T*_*N*_ and *T*_*1*_ represent the recruitment time stamps for the last and first identified MNs, respectively. CSI(*t*) is the cumulative spike train excluding the first recruited MU. The gain *G* was derived from the rheobase currents (minimal electrical current that is necessary to elicit an action potential) of the first and last identified MNs, denoted as *I*_*N*_ and *I*_*1*_, respectively. It is important to note that the CSI, computed from a subset of the MN pool, does not capture the firing activity of the MNs already recruited before the smallest identified MN based on HD-EMG decomposition. Consequently, the input current *I(t)* was defined to remain null until the firing activity of the first identified MN commences at *T*_*1*_ [28].

To estimate the rheobase current of *I*_*N*_ and *I*_*1*_, the decomposed MN was mapped to the entire MN pool based on their recruitment threshold *F* (% Force at MVC) using Eq. (2) (28), which is defined as the percentage of the force generated at the MVC at which the MN becomes active. In this mapping, we can identify the recruitment order of each decomposed MN within the entire MN pool. Since decomposition methods can only capture a subset of the total MNs, mapping allows us to infer the relative position of each identified MN in the pool recruitment hierarchy.

2 $$F(j)={k_1}\left( {{k_2}\frac{j}{N}+\Delta _{{\text{F}}}^{{{{\left( {\frac{j}{N}} \right)}^{1.83}}}}} \right),j \in [1;N]$$

where *j* represents the position of each MN in the recruitment sequence, indicating its order within the MN pool. The approximate size *N* of the MN pool for each muscle was determined based on the literature: 400 for TA [28–31], 950 for SOL [29, 30, 32], and 550 for GM [28–31]. The fitting constants *k*_*1*_, *k*_*2*_, and Δ_*F*_ were uniformly set to 0.5, 58.12, and 120 for all muscles, respectively [28]. Subsequently, the rheobase currents *I*_*N*_ and *I*_*1*_ were estimated using a typical distribution of rheobase values within the MN pools for three muscles, as defined by Eq. (3).

3 $$I(j)={w_1} \cdot \Delta _{I}^{{{{\left( {\frac{j}{N}} \right)}^{1.18}}}}$$

where the fitting constants Δ_*I*_ and *w*_*1*_ were assigned values of 9.1 and 3.9, respectively [19, 28].

### MN Modelling

**Fig. 2:**
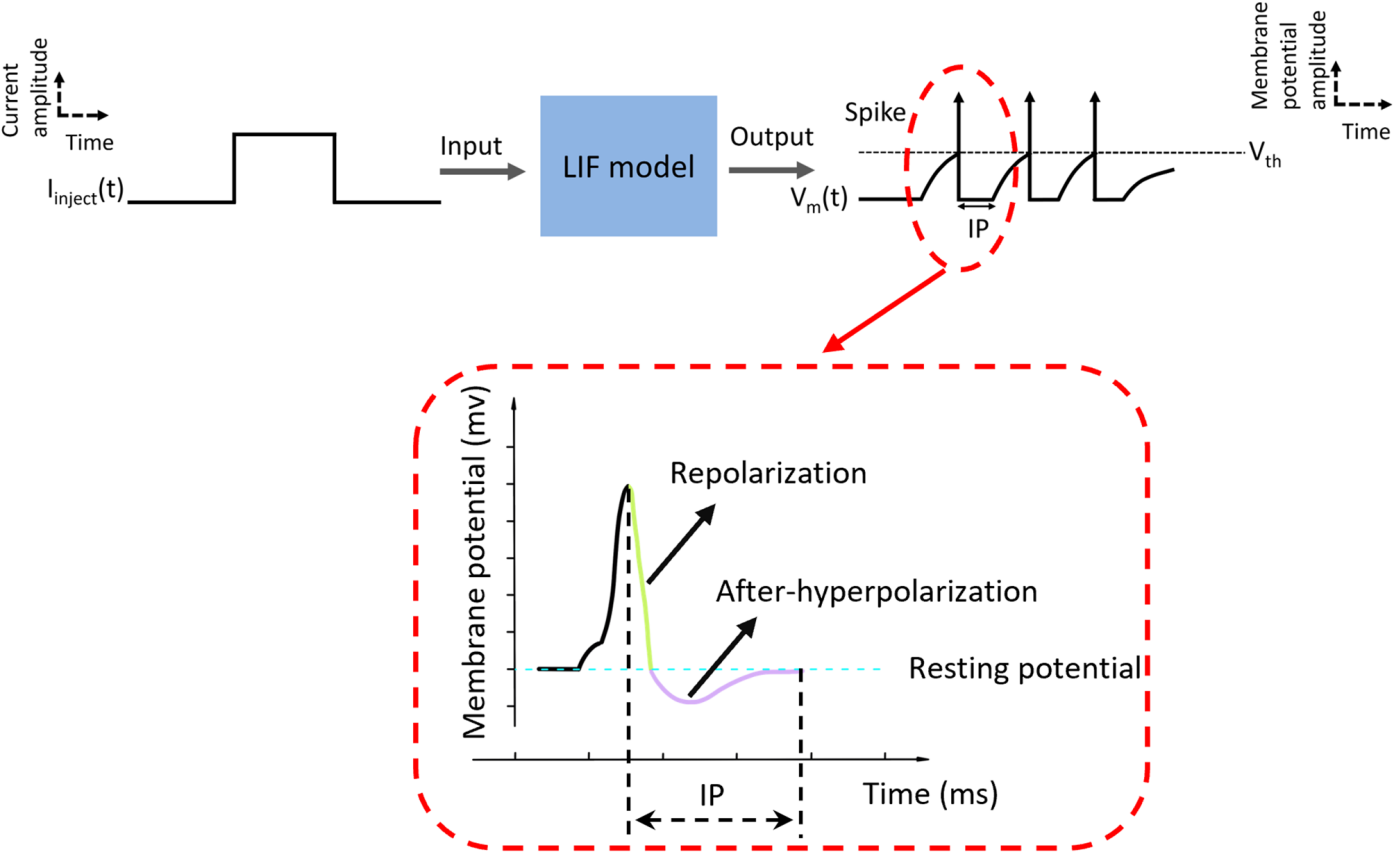
The in silico spike generation using the simplified LIF model. Spikes are generated when the membrane potential V_m_ surpasses the firing threshold due to accumulated input currents. When the membrane voltage V_m_ meets the voltage threshold V_th_, V_m_ is reset and maintained to the membrane resting potential V_reset_ for a time duration IP

To investigate the intrinsic properties of MUs in individuals in vivo, a simplified LIF model was implemented to generate in silico MN spiking trains (Eq. 4). This simplified model was chosen to incorporate only two crucial parameters important for MU recruitment and firing patterns: soma size (Ds) and MN inert period (IP) [28], where IP encompasses both the repolarization and after-hyperpolarization (AHP) periods, as illustrated in Fig. 2. The *τ* represents the time constant, and *R* refers to the membrane resistance. The mathematical relationship underlying this model was derived from an extensive meta-analysis of published experimental data on hindlimb alpha-MNs in adult cats in vivo [19]. In the simplified LIF model, the membrane resting potential *V*_*reset*_ was set to zero, while the membrane threshold *V*_*th*_ was set to 27mV, consistent with typical reported values [19, 28].

4 $$\begin{array}{*{20}{c}}{\tau \frac{{d{V_m}}}{{dt}} = R \cdot I(t) - {V_m}{\text{ }}if:{V_m} > {V_{th}},{\text{ }}{V_m} = 0{\text{ }}for{\text{ }}duration{\text{ }}IP} \\ {\tau = \frac{{2.3 \cdot {{10}^{ - 9}}}}{{Ds_{\text{ }}^{1.48}}},R = \frac{{5.1 \cdot {{10}^{ - 5}}}}{{Ds_{\text{ }}^{2.43}}}} \end{array}$$

### MN parameters and pool properties estimation

The MN parameters Ds and IP were further identified using a genetic algorithm. This optimization process aimed to align the timing of the first spikes between the decomposed spike train (*t*_1, *dec*_) and estimated spike train (*t*_1, *est*_), while minimizing the normalized root-mean-square error (*nRMSE*) between their instantaneous discharge frequencies (IDF) across *n* data points. The sensitivity analysis of Ds and IP on IDF and the first spike time is provided in the supplementary material. The loss function (Eq. 5) was minimized with the support of a genetic algorithm with the following settings: a population size of 50, an elite count of 3, a cross-over rate of 0.8, and a function tolerance of 10^− 6^. The boundaries for Ds were set between 15 and 200, while the boundaries for IP ranged from 0 to 200. The IDF was computed using the Eq. (6), where *t*_*n*_ represents the discharge time of the *n*^*th*^ spike. The IDF was smoothed using a 400 ms Hanning window moving-average filter.

5 $${E_{rror}}=\left| {{t_{1,es}}_{t}{\text{ }} - {\text{ }}{t_{1,dec}}{\text{ }}} \right|+\frac{{\sqrt {\frac{1}{n}\sum\limits_{{i=1}}^{n} {{{\left| {ID{F_{i,{\text{est}}}} - ID{F_{i,{\text{dec}}}}} \right|}^2}} } }}{{\hbox{max} \left( {ID{F_{{\text{dec}}}}} \right) - \hbox{min} \left( {ID{F_{{\text{dec}}}}} \right)}}$$

6 $$ID{F_n}=\frac{1}{{{t_n} - {t_{n - 1}}}}$$

7 $$\left\{ \begin{gathered}Ds = a \times {2.4^{{{\left( {\frac{x}{N}} \right)}^b}}} \hfill \\IP = c \times D{s^d} \hfill \\ \end{gathered} \right\}$$

To estimate the Ds and IP values for MNs that were not directly identified in the decomposition process, the estimated MN parameters Ds and IP corresponding to 20% and 50% MVC levels were combined. Their distributions across the entire MN pool were modelled using Eq. (7) [19]. Specifically, a non-linear least-squares fitting method was applied to determine the fitting coefficients *a*, *b*, *c*, and *d*. *x* denotes the position of the MNs in the pool, ranging from the minimum to the maximum values *j* in Eq. (2). The coefficient of determination (*r*^*2*^) was used to evaluate the goodness of fit for the model. After obtaining the distributions of Ds and IP parameters across the entire MN pool, these distributions were then used to calculate the extended MN pool parameters within the minimal and maximal decomposed MN index in the pool.

### Validation

To validate the generalization capability of the simplified LIF model, the performance of the model was evaluated from a subset of participants (five participants with SCI and five able-bodied participants) who had the same number of decomposed MUs across four repetitions, with one randomly selected trial per participant. We first estimated the MN parameters Ds and IP from the first two consecutive repetitions using the previously described steps. These parameters were then used in the simplified LIF model to simulate spike trains for the last two consecutive repetitions. Whether the simulated spike train matched the decomposed spike trains in the last two consecutive repetitions was evaluated. The gain *G* was kept constant for input current estimation, as the MNs remained consistent across these repetitions. Specifically, we calculated the *nRMSE* of IDF between the decomposed and simulated spike trains.

### Statistical analysis

The model estimated outcome parameters include Ds, IP, and mean firing rate for each muscle and each participant. The average error between the IDF of decomposed and estimated spike trains was quantified using *nRMSE*, while the error of the mean firing rates of the decomposed and estimated spike trains was quantified using *RMSE*. A lower RMSE indicates better alignment between the estimated and decomposed spike trains. The Shapiro-Wilk test was used to examine whether errors follow the normal distribution. If the normality assumption was satisfied, the Welch two-Sample t-test to compare the difference between different groups was conducted with a significant level of *p* < 0.05. Alternatively, the Mann-Whitney U test was applied when normality was not satisfied. To evaluate differences in estimated parameters Ds and IP both within and between groups at different contraction levels (20% MVC and 50% MVC), and to evaluate the similarity between decomposed and estimated MN pool parameters, the linear mixed-effects regression (using the lme4 package in R) was exploited. It helped account for the variability in the number of identified MUs for each participant. In this model, groups were treated as a fixed effect, while participants and the number of decomposed MUs per participant were treated as random effects. The statistical analysis was conducted using R version 4.3.3 on a Windows platform.

## Results

To ensure a reliable mapping to the MN pool, data of the muscle was included only if at least three MNs could be decomposed. Therefore, further analysis for the SOL muscle included twenty-one participants with SCI and seventeen controls, for the GM muscle sixteen participants with SCI and eleven controls, and for the TA muscle twenty-two participants with SCI and sixteen control participants. Consequently, the results were presented based on subsets of participants in two groups, with a muscle-dependent sample size.

### Intra-subject reliability

**Fig. 3:**
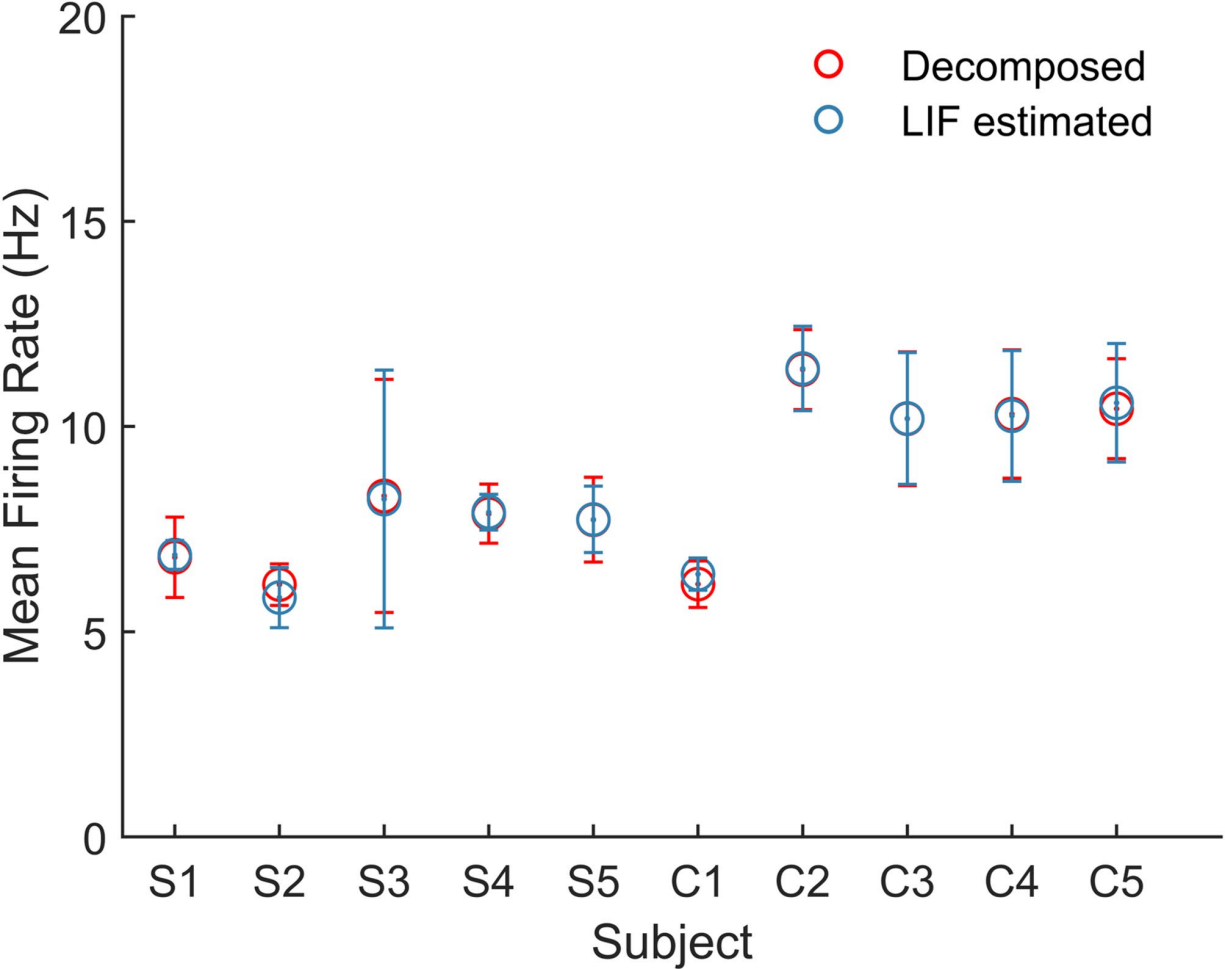
Validation results for the simplified LIF model. MN parameters Ds and IP were estimated from the first two consecutive repetitions and then used as inputs for the simplified LIF model to generate LIF estimated spike trains. These LIF estimated spike trains were compared with decomposed spike trains from the last two consecutive repetitions. The mean firing rates of decomposed and LIF estimated spike trains were computed for five participants with SCI (S1-S5) and five non-disabled control participants (C1-C5), with error bars indicating the standard deviation

When the LIF model was used to validate intra-subject trials under the same contraction level, the model could effectively capture overall spike characteristics of decomposed MUs in both groups, achieving a similar *nRMSE* in IDF for both groups (SCI: 0.342 ± 0.08, control: 0.306 ± 0.04) and a low *RMSE* for the mean firing rates (*RMSE SCI*: 0.646 ± 0.19 Hz, control: 0.614 ± 0.164 Hz) (Fig. 3).

### Decomposed MN parameter estimation

**Fig. 4:**
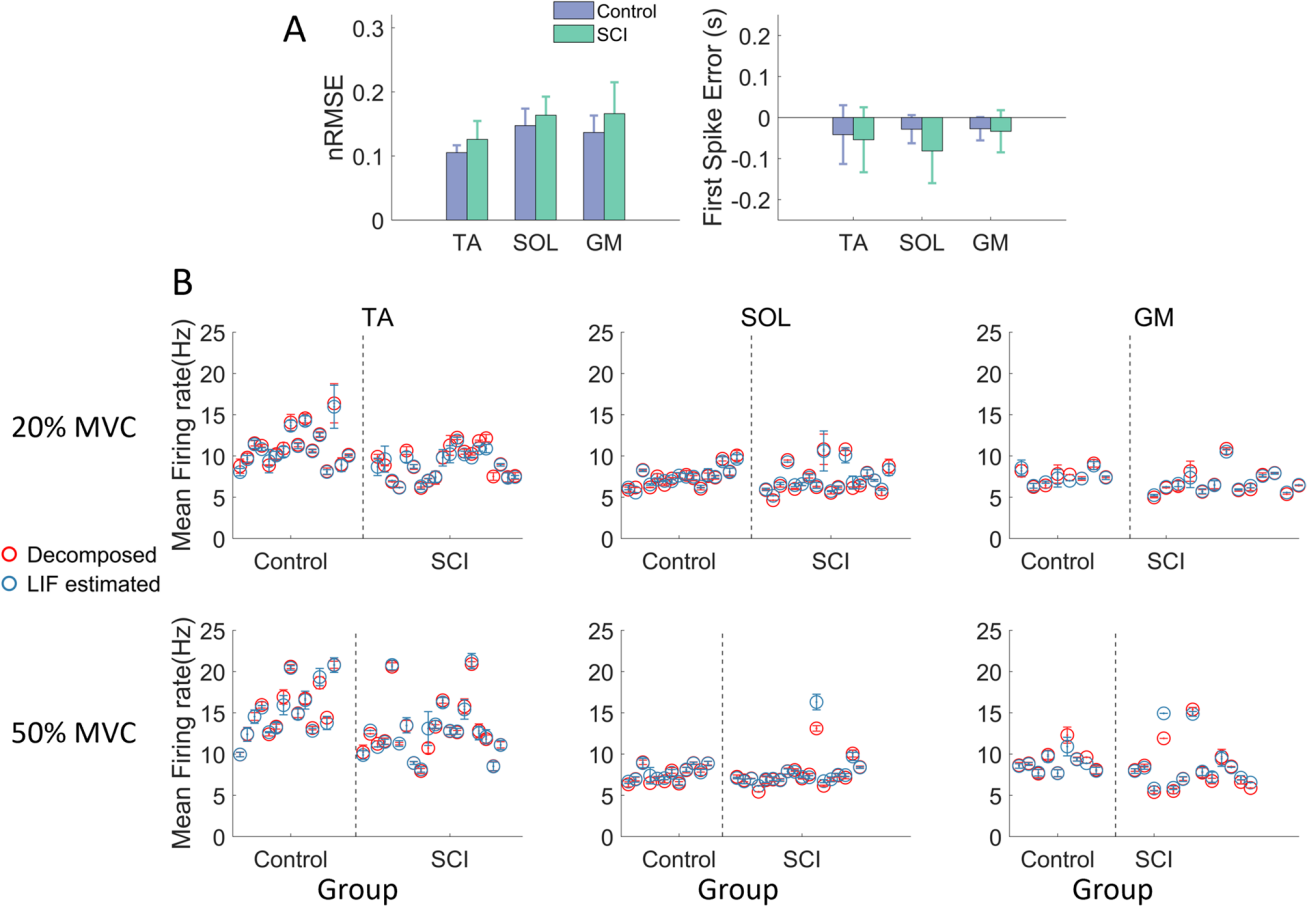
Comparison of decomposed and estimated MU spike trains. **A**: MN parameters were estimated using a genetic algorithm by aligning the timing of the first spikes between decomposed and estimated spike trains while minimizing the normalized root mean square error (nRMSE) of their instantaneous discharge frequencies. The resulting cost function values are shown. **B**: The estimated MN parameters were then used to generate in silico spike trains with a simplified LIF model, and their mean firing rates were compared with those of the decomposed spike trains. Mean firing rates are presented for each muscle in the SCI and control groups at contraction levels of 20% and 50% MVC. Each circle represents the mean firing rate of one participant, with error bars indicating the standard deviation.

**Table 1:** Mean firing rates and RMSE for each muscle in the SCI and control groups. The mean firing rate was calculated per subject, then averaged across each group (SCI and control) for each muscle at two contraction levels. The RMSE was determined between the mean firing rates of decomposed and estimated Spike trains, and the results were similarly averaged across each group for each muscle and contraction level

		20% MVC			50%MVC		
		TA	SOL	GM	TA	SOL	GM
Control	Decomposed (Hz)	11.14 ± 2.29	7.46 ± 1.20	7.53 ± 0.92	15.34 ± 3.16	7.57 ± 1.03	9.13 ± 1.44
	Estimated (Hz)	10.93 ± 2.22	7.42 ± 1.08	7.44 ± 0.84	15.20 ± 3.19	7.67 ± 0.86	8.84 ± 1.04
	RMSE (Hz)	0.98 ± 0.57	0.54 ± 0.16	0.75 ± 0.43	1.14 ± 0.48	0.77 ± 0.48	0.70 ± 0.53
SCI	Decomposed (Hz)	9.06 ± 1.99	7.11 ± 1.79	6.76 ± 1.57	12.73 ± 3.46	7.61 ± 1.68	8.24 ± 2.81
	Estimated (Hz)	8.68 ± 1.83	7.18 ± 1.50	6.78 ± 1.37	12.86 ± 3.48	7.83 ± 2.26	8.57 ± 2.99
	RMSE (Hz)	1.03 ± 0.59	0.82 ± 0.70	0.56 ± 0.24	1.38 ± 0.97	1.02 ± 1.63	1.09 ± 1.19

The overall *nRMSE* of model-predicted spike trains remained consistently below 0.2 for all muscles in both groups, with the TA in the control group showing a significantly smaller error compared to the SCI group (TA: *p* < 0.01, SOL: *p* = 0.07, and GM: *p* = 0.06) (Fig. 4A). Specifically, the control group showed a smaller first spike error in SOL compared to the SCI group (TA: *p* = 0.19, SOL: *p* < 0.01, and GM: *p* = 0.87). The mean firing rates of the estimated spike trains match well with the decomposed MNs (Table 1). No significant difference was observed in RMSE between the two groups at either 20% MVC (TA: *p* = 0.81, SOL: *p* = 0.69, and GM: *p* = 0.08) or 50% MVC (TA: *p* = 0.95, SOL: *p* = 0.19, and GM: *p* = 0.16) (Fig. 4B).

**Fig. 5:**
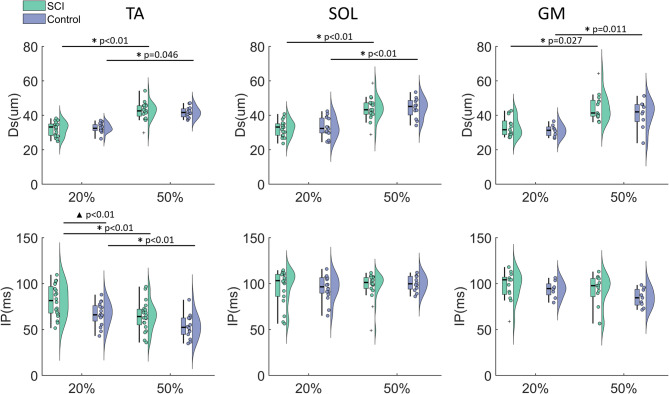
Comparison of MN parameters between the SCI and control groups across muscles and contraction levels. MN parameters (Ds and IP) were estimated from the MNs in the TA, SOL, and GM muscles at 20% MVC and 50% MVC. Each dot represents the mean Ds or IP of one participant, with the boxplots on the left and their distributions shown on the right. Asterisks (∗) indicate significant differences in the parameter within the same group between different contraction levels, while triangles (▲) denote significant differences in the parameter between the SCI and control groups at the same contraction levels

For both groups, the mean Ds was significantly larger at 50% of MVC compared to the 20% MVC for all muscles (Fig. 5). However, no significant between-group differences in the Ds were observed in either muscle at 20% or 50% MVC (Table 2).

**Table 2:** Between-group comparison of Ds and IP parameters in each group for each muscle. Values were presented as mean ± standard deviation. The null hypothesis assumes no significant difference in these parameters between the SCI and control groups. Statistical analysis was performed using a linear mixed-effects regression model

Groups		TA			SOL			GM		
	MVC	Control	SCI	*p*-value	Control	SCI	*p*-value	Control	SCI	*p*-value
Ds(µm)	20% MVC	32.67 ± 2.67	32.13 ± 3.99	0.65	32.81 ± 5.65	32.46 ± 4.59	0.39	31.10 ± 3.34	33.25 ± 5.54	0.93
	50% MVC	41.72 ± 3.04	42.51 ± 5.04	0.11	44.25 ± 5.81	43.94 ± 6.44	0.89	40.68 ± 8.26	44.77 ± 7.90	0.25
IP(ms)	20% MVC	66.74 ± 12.52	81.32 ± 17.41	< 0.01	95.37 ± 13.69	96.29 ± 19.25	0.94	93.72 ± 8.98	97.21 ± 15.98	0.41
	50% MVC	53.35 ± 13.02	64.55 ± 16.08	0.08	100.10 ± 8.27	97.54 ± 15.02	0.60	84.60 ± 9.84	91.78 ± 18.45	0.30

At 20% MVC, the mean IP in TA was significantly longer in the SCI group compared to controls (Control: 66.74 ± 12.52 ms, SCI: 81.32 ± 17.41 ms, *p* < 0.01) (Fig. 5), but no significant between-group differences were found in the SOL and GM (Table 2). At 50% MVC, no difference in the mean IP was observed in either muscle. However, there was a trend that the mean IP in TA remained shorter in the SCI group compared to the control group (*p* = 0.08). Compared to 20% MVC, the TA showed a significantly shorter mean IP at 50% MVC for both groups (Control: *p* < 0.01, SCI: *p* < 0.01). However, no differences can be observed in SOL or GM, although the control group showed a trend toward a shorter mean IP in the GM at 50% MVC compared to 20% MVC (*p* = 0.065).

### Extended MN pool

**Fig. 6:**
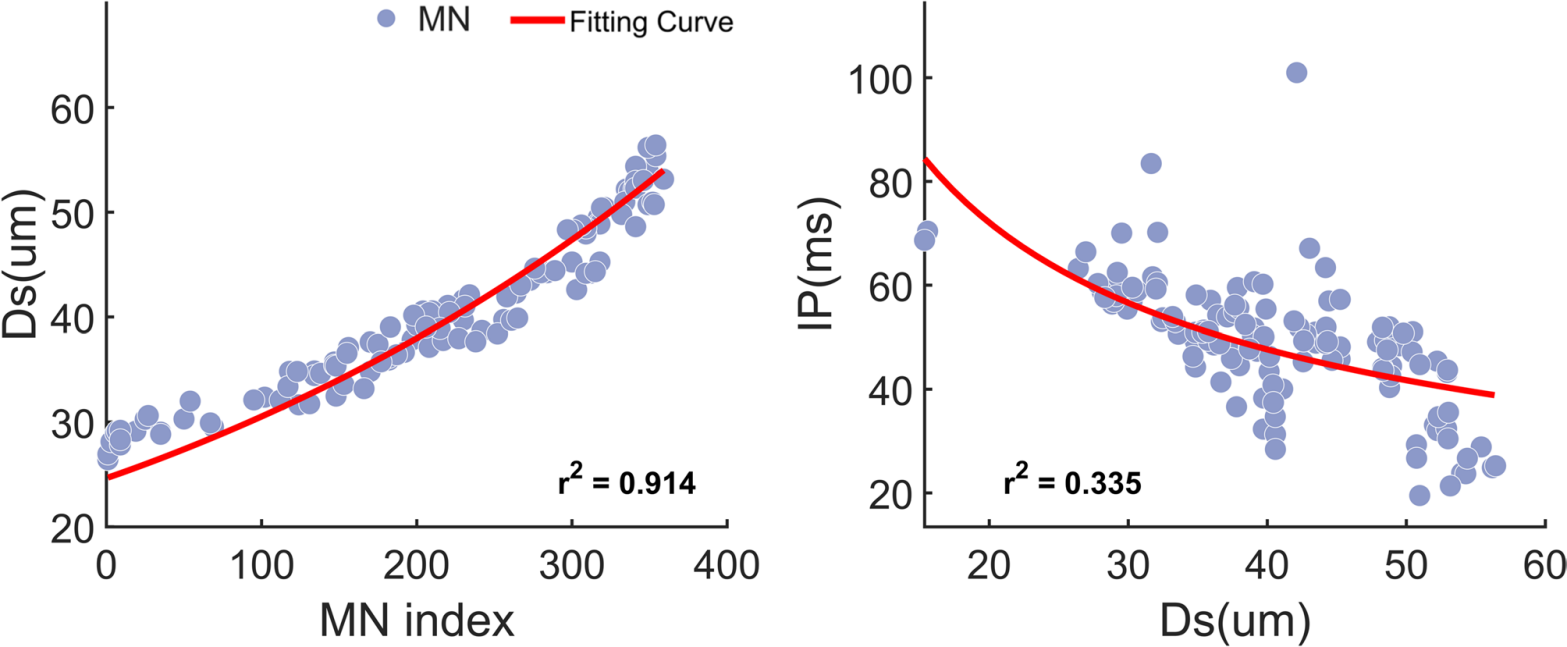
The MN pool fitted curve for Ds and IP parameters. The parameters were estimated from one example control participant in the TA muscle. Each dot represents an individual MN, with all MNs from this participant at 20% and 50% MVC pooled together. The MN index denotes the position j of each MN in the MN pool calculated by Eq. (2). A non-linear least-squares fitting method was applied to model the relationship. The coefficient of determination (*r*^2^) was used to evaluate the goodness of fit

Figure 6 illustrated a representative example of using decomposed MN parameters to estimate their distributions across the entire MN pool. A good fitting for Ds can be observed in both groups across all muscles (Table 3). However, the estimation of the IP was based on the mathematical relationship between Ds and IP, resulting in relatively low *r*^*2*^ values in both SCI and control groups (Table 3).

**Table 3:** The average of fitting coefficients A, B, C, and d for each muscle in all groups. These coefficients were determined using a non-linear least-squares fitting method, applied to the model in Eq. (7). r^2^ is the coefficient of determination. Values were presented as mean ± standard deviation

Groups	TA			SOL			GM		
Ds	*a*	*b*	*r* ^*2*^	*a*	*b*	*r* ^*2*^	*a*	*b*	*r* ^*2*^
SCI	23.79 ± 5.48	0.72 ± 0.62	0.71 ± 0.18	23.49 ± 5.98	0.64 ± 0.77	0.60 ± 0.18	23.48 ± 5.36	0.54 ± 0.47	0.59 ± 0.24
Control	21.93 ± 4.76	0.66 ± 0.63	0.73 ± 0.15	23.39 ± 5.96	0.71 ± 0.63	0.50 ± 0.21	23.75 ± 7.14	1.40 ± 1.38	0.65 ± 0.23
IP	*c*	*d*	*r* ^*2*^	*c*	*d*	*r* ^*2*^	*c*	*d*	*r* ^*2*^
SCI	485.72 ± 490.15	-0.44 ± 0.29	0.20 ± 0.13	2675 ± 10,719	-0.25 ± 0.43	0.10 ± 0.16	290.45 ± 197.38	-0.25 ± 0.22	0.08 ± 0.09
Control	498.46 ± 283.98	-0.55 ± 0.22	0.21 ± 0.13	190.62 ± 102.12	-0.16 ± 0.17	0.06 ± 0.08	8205 ± 26,061	-0.49 ± 0.55	0.16 ± 0.12

The extended MN pool parameters were calculated and compared with the decomposed MN parameters. It was shown that the extended MN pool parameters consistently matched the range of the decomposed MN parameters (Table 4) with no significant differences observed (*p* > 0.05 for all groups and all muscles).

**Table 4:** Comparison of MN parameters between decomposed and estimated MN pool. The comparison aims to evaluate the differences in these parameters between decomposed and the estimated MN pool for both SCI and control groups across different muscles (TA, SOL, GM). Values are presented as mean ± standard deviation. The null hypothesis assumes no significant differences between the decomposed MNs and the MN pool for each muscle and MN parameter

Groups		TA			SOL			GM		
		Decomposed	Pool	*p*-value	Decomposed	Pool	*p*-value	Decomposed	Pool	*p*-value
SCI	Ds(µm)	38.05 ± 5.43	39.59 ± 4.93	0.46	38.44 ± 6.00	40.89 ± 5.97	0.53	38.71 ± 5.37	41.24 ± 5.56	0.32
	IP(ms)	72.10 ± 16.59	70.24 ± 17.55	0.68	96.94 ± 16.63	95.30 ± 17.96	0.78	96.21 ± 15.94	94.32 ± 16.87	0.75
Control	Ds(µm)	36.64 ± 3.10	37.49 ± 2.67	0.45	37.17 ± 6.92	38.60 ± 6.55	0.74	36.92 ± 7.94	36.20 ± 7.07	0.93
	IP(ms)	61.61 ± 12.79	60.19 ± 12.86	0.89	96.95 ± 10.00	95.94 ± 10.68	0.76	86.95 ± 8.98	85.21 ± 11.54	0.46

## Discussion

In this study, we proposed a novel method that integrates HD-EMG decomposition and spike neuron model to estimate personalized, physiologically meaningful MU parameters in vivo in major ankle dorsi- and plantarflexors. In addition, we investigated potential alternations in these parameters in persons with SCI. This is the first study to use such an integrated approach to examine MU electrophysiological changes in persons with SCI in vivo, offering new insights into neural adaptations following impairment. The results revealed that MU properties were altered in individuals with SCI with a significantly prolonged IP in the TA muscle. However, no significant differences were observed in either ankle plantarflexor. The estimated parameters were then used to create a complete subject-specific in silico MN pool, thereby providing a comprehensive characteristic description for MN pool parameter distribution and enabling the inference of MU properties not directly accessible through decomposition. Our proposed method provided a non-invasive and reliable approach to monitor MN behavior in vivo, offering deeper insights into MN adaptation following impairment.

The simplified LIF model effectively captured essential MN characteristics crucial for force generation and modulation and demonstrated great generalization ability for capturing in vivo decomposed MN firing patterns across different muscles and varying levels of isometric contraction. The LIF model simplified the complex biophysical neuron dynamics into two key parameters: Ds, and IP, based on relationships between electrophysiological parameters of MUs derived from spinal MNs in animal models. The Ds regulates the recruitment threshold, with smaller MNs experiencing greater membrane potential changes than larger ones under the same synaptic currents according to Ohm’s law [33]. The IP, on the other hand, governs firing patterns, with higher IP leading to a longer duration of hyperpolarization and delaying the neuron’s ability to reach the firing threshold [28, 34]. Based on established electrophysiological relationships derived from spinal MNs in animal models, the estimated parameters for Ds and IP in our study fall within the ranges reported in the literature [19]. Our finding revealed that participants with SCI exhibited a prolonged inert period in the TA muscle, while no changes were observed in the SOL and GM muscles. In this study, the IP encompassed both the repolarization and AHP phases, with the repolarization duration typically lasting only a few milliseconds [35]. Therefore, the IP duration was predominantly determined by the AHP duration, a key regulator of MN firing rates. The AHP is primarily shaped by potassium channel activation and potassium ion efflux, which govern the hyperpolarization phase. Therefore, in individuals with SCI, a prolonged IP may result from altered potassium channel’s function or expression, potentially influenced by changes in the post-injury ionic environment [36]. The extended IP observed in the TA muscle aligns with previous findings, which indicated that the AHP duration is a key MU parameter affected by neurological conditions such as stroke and SCI based on both human [34, 37] and animal studies [38]. A prolonged IP in MNs reduces MN firing rates, limiting the TA muscle’s capacity to generate rapid, sustained contractions, and contributing to muscle weakness [1, 39]. Several factors, including medication use, spasticity, and daily activity levels, can influence MU properties in SCI participants. For instance, baclofen, a commonly used anti-spasticity medication, has been shown to inhibit MN excitability [40, 41], which may potentially prolong the IP. In contrast, participation in rehabilitation exercises may enhance MU and excitability. Future research should consider these variables to better understand MU properties after SCI.

In contrast, the SOL and GM muscles showed no significant changes in the IP parameter in persons with SCI, potentially due to differences in muscle fiber composition, functional roles, or compensatory adaptations not fully addressed in this study. The TA, which contains a higher proportion of fast-twitch MUs, exhibited a noticeable reduction in IP with increasing contraction intensity, likely attributable to the faster firing capabilities and shorter AHP durations of its MNs. Conversely, the SOL and GM, which have a higher proportion of slow-twitch fibers, are more involved in postural control and sustained contractions, may rely on more stable and prolonged recovery dynamics, resulting in relatively shorter and unchanged IP values across contraction levels [42, 43]. These muscle-specific differences highlight the importance of considering both neural and muscular characteristics when interpreting IP alternations in SCI across different muscles. Although the simplified LIF model effectively captures key aspects of MN behavior such as recruitment and firing patterns, it does not account for more complex electrophysiological processes. For instance, ionic conductance variability and dendritic processing are not represented in the simplified LIF model, which may limit its ability to fully capture MN firing behavior under pathological conditions such as SCI.

The intra-subject evaluation showed that the proposed model can accurately reproduce the IDF and mean firing rates of MUs in both SCI and control groups, confirming its ability to capture key firing patterns of MUs with high robustness, despite a slightly better overall fitting in the control group. This between-group difference in the fitting performance may be attributed to irregular firing behavior observed in the participants with SCI, where MUs may fire more erratically and less synchronously, leading to delays and inconsistencies that make it harder for the model to capture the exact firing dynamics [1, 44]. However, it is worth noting that the model always predicted the first spike slightly earlier than the actual firing time. This was likely because the optimization aimed to align the first spike timings while matching IDF across the entire spike train. The optimization prioritized overall IDF (interval between spikes) accuracy over the first spike alignment, resulting in a small systematic bias. The validation in this study was conducted using consecutive trials under the same experimental conditions, which may limit the ability to explore potential variability in the model’s performance across different experiment conditions. Future studies could address this by introducing variations in experimental conditions, such as altering ankle angles while maintaining consistent contraction levels across trials, to enable a more rigorous model validation.

A significantly positive relationship between the size of MNs, i.e., soma size Ds and contraction levels was observed in both groups. Specifically, MNs with larger Ds were recruited more prominently during higher contraction levels. This observation aligned with Henneman’s size principle [45], suggesting that MUs were recruited in order of increasing size as force requirements escalated. Ds plays a crucial role in determining the recruitment threshold of MNs. Larger MNs with greater soma size and surface area, therefore, have smaller input resistance, requiring stronger synaptic input to reach their firing threshold. Consequently, they were recruited later in a contraction, primarily during high force demands.

An extended MU pool could be created by identifying the distribution of the decomposed MN parameters, effectively preserving the characteristics of the originally identified MNs while increasing the total number. The distributions of MU parameters within the pool were estimated based on fitting functions derived from animal models [19]. This approach ensured biological plausibility and accuracy in modeling the distribution of MN properties within the pool. In our study, the distribution of Ds was effectively captured across the MN pool. However, the adopted fitting function could not fully account for the relationship between Ds and IP, as IP could be influenced by a range of factors such as muscle fiber type composition and pathological conditions, contributing to the greater physiological variability of IP across the MN pool. In addition, our mapping approach also depended on the recruitment thresholds of the decomposed MUs. Since the first recruited decomposed MN might be recruited much later in the actual pool sequence, as only a small subset of the MNs were captured through decomposition. Also, the decomposed MNs were assigned a recruitment index within the pool and both groups were assigned the same MN pool size and distribution parameters (Eq. 2). However, it has been reported that SCI could lead to fewer MNs [46, 47], therefore a smaller pool size. This discrepancy could potentially lead to an overestimation of the recruitment index of the decomposed MN in the SCI group. Future studies incorporating subject-specific estimations of MN pool size, such as the Motor Unit Number Index technique [48] would facilitate more precise estimations of MU parameters with a varied pool size.

It was worth mentioning that our proposed integrated approach relies on the availability of decomposed in vivo MNs. Compared to the TA muscle, fewer MNs were identified in the SOL and GM muscles, likely due to factors such as their functional anatomy [8]. The deeper location and greater size of the SOL and GM muscles could result in more overlapping action potentials, making it challenging to accurately decompose and identify individual MUs. A different decomposition algorithm, peel-off convolution kernel compensation method [49] has been reported to significantly increase the number of identified MNs. Future studies with such an approach could probably enhance the statistical power in evaluating MU properties in non-superficial and large muscles, which are otherwise difficult to decompose. Another limitation of this study is the small sample size. Future research with larger sample sizes could provide more robust and generalized conclusion into MU behavior in individuals with SCI. While the simplified LIF model enables non-invasive estimation of MU properties, it simplifies biophysical complexities, and estimates of Ds and IP are model-based approximations. Independent empirical electrophysiological data, such as direct measurements of MN properties through invasive recordings, would provide more robust validation. However, surgical access to individual MNs is not practical in human studies, particularly in clinical populations. Therefore, the estimated parameters should be viewed as functionally representative rather than anatomically precise.

In conclusion, this study presented an integrated approach that combined HD-EMG decomposition and MN modelling to effectively estimate MU parameters in vivo. The model demonstrated good generalization and high accuracy in replicating in vivo MN firing patterns for the first time in both able-bodied participants and those with SCI. A significantly prolonged IP was observed in the TA muscle in persons with SCI indicating MU in the TA muscle experience a longer recovery phase before they can be re-excited. In contrast, the SOL and GM muscles showed no significant changes in either parameter, possibly due to their different functional anatomy. However, the limited number of decomposed MUs constrained our ability to fully interpret these results in SOL and GM. Additionally, a larger MN pool was reconstructed using the estimated parameters, providing valuable insights into MU recruitment and distribution within the pool. This comprehensive analysis enhances our understanding of MU property alterations in persons with SCI and highlights the potential of such an approach for future investigating into MU-level adaptations following therapeutic intervention.

## Electronic supplementary material

Below is the link to the electronic supplementary material.

Supplementary Material 1

## Data Availability

No datasets were generated or analysed during the current study.

## Abbreviations

AHP: After-hyperpolarization
CSI: Common synaptic input
Ds: Soma size
GM: Gastrocnemius medialis
HD-EMG: High-density electromyography
IDF: Instantaneous discharge frequency
IP: Inert period
LIF: Leaky integrate-and-fire
MN: Motor neuron
MU: Motor unit
MVC: Maximal voluntary contraction
nRMSE: normalized root-mean-square error
r^2^: Coefficient of determination
RMSE: Root-mean-square error
SCI: Spinal cord injury
SOL: Soleus
TA: Tibialis anterior
