# Supplementary material for: In vivo estimation of motor unit intrinsic properties in individuals with spinal cord injury
Source: J Neuroeng Rehabil. 2025 Jun 5;22:128. doi: 10.1186/s12984-025-01659-z (PMC12139106; doi:10.1186/s12984-025-01659-z)
Supplement: Supplementary file 1 — Supplementary Material 1 [file 12984_2025_1659_MOESM1_ESM.docx]

**Sensitivity Analysis of Model Parameters**

To evaluate the robustness of the model and examine the impact of parameter variations on the output spike trains, we performed a sensitivity analysis of two key model parameters: soma size (Ds) and inert period (IP). The analysis was conducted as follows:

1. **Setup:** We applied a fixed input current to the simplified LIF model with representative MN model parameters (Ds = 30 µm; IP = 80 ms), which produced a physiologically plausible spike train. We then systematically varied one parameter at a time:

2. **Parameter Variations:**

- **Ds:** Ds was varied from 90% to 110% of its baseline value in 2% increments, while keeping IP constant at 80 ms.
- **IP:** IP was varied similarly from 90% to 110% of its baseline value in 2% increments, with Ds held constant at 30 µm.

3. **Analysis:** For each variation in parameter values, we generated new MN spike trains, calculated their instantaneous discharge frequency (IDF), and quantified the difference from the baseline IDF using normalized RMSE (nRMSE). The normalization was performed by dividing the difference in IDF by the range of the baseline IDF (i.e., max(IDF) – min(IDF)). In addition to IDF analysis, we also evaluated the sensitivity of each parameter to the first spike time (t_1,base_ - t_1,altered_, means the first spike time from initial values - the first spike time from adjusted values), which allowed us to assess how changes in Ds and IP influence motor neuron recruitment timing.

4. **Results:** The sensitivity analysis revealed that:

- **Ds:** Ds strongly influenced the nRMSE, primarily due to its effect on MN recruitment. A mismatch in the timing of the first spike time leads to a large overall error in the IDF.
- **IP:** IP did not affect MN recruitment but modulated MN firing patterns, leading to changes in nRMSE. However, the sensitivity of IP was smaller compared to Ds.

(A) (B)

*Figure 1. Sensitivity analysis of model parameters. (A) The effect of changes in Ds and IP on the nRMSE of the instantaneous discharge frequency (IDF). The x-axis shows the variations in Ds and IP individually, with one parameter changed at a time while the other is held constant. The y-axis represents the corresponding changes in nRMSE. (B) The effect of changes in Ds and IP on the first spike time error (t_1,base_ - t_1,altered_). The x-axis shows the variations in Ds and IP, and the y-axis represents the resulting changes in the first spike time error.*

*Table 1. Demographic and clinical characteristics of participants with spinal cord injury (SCI). The table presents participant ID, weight (kg), height (mm), age (years), gender (F-female, M-male), time since injury (month), and injury level.*

| SCI id | Weight (kg) | Height (mm) | Age (year) | Gender | Time since injury (month) | Injury level |
| --- | --- | --- | --- | --- | --- | --- |
| 1 | 75 | 1730 | 55 | F | 132 | T3 |
| 2 | 86.9 | 1565 | 58 | F | 7 | T5 |
| 3 | 111.8 | 1755 | 53 | F | 7 | T5 |
| 4 | 73.7 | 1749 | 61 | M | 23 | T6 |
| 5 | 114.5 | 1788 | 65 | M | 24 | T11 |
| 6 | 84 | 1640 | 47 | F | 29 | T6 |
| 7 | 103.1 | 1850 | 53 | M | 168 | T4 |
| 8 | 95.3 | 1794 | 73 | M | 18 | T10 |
| 9 | 64 | 1600 | 51 | F | 42 | T6 |
| 10 | 83.6 | 1870 | 32 | M | 24 | T6 |
| 11 | 95 | 1840 | 69 | M | 120 | T10 |
| 12 | 64 | 1650 | 73 | M | 24 | T4 |
| 13 | 58.7 | 1709 | 68 | F | 12 | T4 |
| 14 | 52.9 | 1592 | 60 | F | 180 | L1 |
| 15 | 62.4 | 1680 | 49 | F | 18 | L5 |
| 16 | 97 | 1885 | 30 | M | 36 | T4 |
| 17 | 51.9 | 1632 | 73 | F | 144 | T11 |
| 18 | 74.8 | 1730 | 42 | M | 204 | T7 |
| 19 | 80.8 | 1805 | 54 | M | 10 | C2* |
| 20 | 68.1 | 1630 | 56 | F | 16 | C8* |
| 21 | 80 | 1746 | 35 | M | 72 | L3 |
| 22 | 90 | 1758 | 45 | M | 60 | T11 |
| 23 | 95.7 | 1754 | 68 | M | 144 | T7 |

_*_ Minor sensory deficits at the cervical level were allowed, as long as the motor impairment was consistent with paraplegia, meaning it involved the thoracic level or below.

*Table 2. The average parameter constant gain G for each muscle in all groups (mean ± standard deviation).*

| Gain | TA | | SOL | | GM | |
| --- | --- | --- | --- | --- | --- | --- |
|  | 20% | 50% | 20% | 50% | 20% | 50% |
| SCI | 75.11±67.78 | 161.33±89.85 | 133.33±95.80 | 431.50±241.03 | 237.28±206.25 | 469.71±475.67 |
| Control | 83.16±71.28 | 156.47±53.17 | 198.62±226.35 | 521.94±228.79 | 107.82±67.13 | 228.23±138.22 |

*Table 3. P-values for the comparison of parameters Ds and IP between 20% and 50% MVC in each muscle. The null hypothesis assumes no significant difference in these parameters between the 20% and 50% MVCs. Statistical analysis was performed using a linear mixed-effects regression model.*

| Muscle | | TA | SOL | GM |
| --- | --- | --- | --- | --- |
| MVC | | 20% VS 50% | 20% VS 50% | 20% VS 50% |
| Ds(µm) | Control | p=0.046 | p<0.01 | p=0.011 |
|  | SCI | p<0.01 | p<0.01 | p=0.027 |
| IP(ms) | Control | p<0.01 | p=0.89 | p=0.065 |
|  | SCI | p<0.01 | p=0.36 | p=0.43 |
